# Non-destructive label-free automated identification of bacterial colonies at the species level directly on agar media using digital holography and convolutional neural network algorithms

**DOI:** 10.1128/spectrum.00080-26

**Published:** 2026-07-13

**Authors:** Prisca Perlemoine, Jordan Belissard, Bruno Burtschell, Nassim Halli, Luc Martin, Camille Brunet, Maxime Gougis, Patrick Schiavone, Yvan Caspar

**Affiliations:** 1 BAIO-DX, Grenoble, France; 2 Laboratoire de bactériologie hygiène hospitalière, Centre Hospitalier Universitaire Grenoble Alpes 36724, Grenoble, France; 3 Univ. Grenoble Alpes, CNRS, CHU Grenoble Alpes, CEA, IBS, Grenoble, France; Ascension St John Hospital, Detroit, Michigan, USA

**Keywords:** identification method, automation, digital holographic imaging, bacteria, artificial intelligence, machine learning, convolutional neural network, microbiology

## Abstract

This study aimed to establish a thorough proof of concept for an innovative, fully automated, non-destructive, and label-free approach for the identification of bacterial colonies directly on agar plates, integrating digital holography with artificial intelligence, and to evaluate its performance. High-resolution holographic images of individual bacterial colonies grown on translucent brain-heart agar plates were captured every 30 min over an 18-h incubation period (530 MPx per full plate). Imaging was performed using a large field 1× magnification system, a partially coherent LED light source, and a high-resolution complementary metal-oxide-semiconductor (CMOS) sensor. A database containing 49,490 digital holograms of individual colonies from 276 clinical strains belonging to 10 of the most prevalent pathogenic bacterial species was used to train the convolutional neural network (CNN). Prediction accuracy was further enhanced by incorporating information across different phylogenetic levels. The performance of the BAIO-DX system was evaluated on 232 strains from the 10 species included in the training data set, as well as 64 strains from 8 species not included in the training data set. For the species included in the training data set, this new method identified 86.6% of the strains at the species level, with a positive-percent agreement of 96.5%. An additional 48% of the strains not identified at the species level could be correctly classified at the genus level through phylogenetic interpretation. These results validate this innovative approach as a candidate for a fully automated, non-destructive, and label-free solution for bacterial identification in clinical microbiology laboratories.

**IMPORTANCE:**

Identification of pathogenic bacteria in clinical laboratories is traditionally performed using culture-based methods, such as matrix-assisted laser desorption/ionization-time-of-flight (MALDI-TOF) mass spectrometry or biochemical assays. Although recent advances in automation and artificial intelligence (AI) applied to agar plate imaging have reduced manual workload, bacterial identification remains time-consuming and labor-intensive. Here, we present a fully automated, non-destructive, and label-free identification method of bacterial colonies directly on agar plates, combining digital holography and convolutional neural network (CNN) algorithms. After training the system with 276 strains belonging to 10 of the most frequent pathogenic species, the BAIO-DX solution was able to identify 86.6% of new strains at the species level, on a 232-sample test set, achieving a positive-percent agreement of 96.5%. These thorough proof of concept shows that advanced imaging methods with AI-driven analysis can enable reliable, fully automated identification of a substantial proportion of clinically relevant bacteria, offering significant potential to streamline and enhance diagnostic workflows in clinical microbiology.

## INTRODUCTION

Clinical bacteriology has long relied on manual, labor-intensive culture-based methods. Over the past two decades, major advances, such as matrix-assisted laser desorption/ionization-time-of-flight (MALDI-TOF) mass spectrometry and Microbiology Laboratory Automation (MLA) systems, have revolutionized microbial identification and workflow efficiency (1). Through robotics and software developments, MLA systems associate inoculation and streaking unit(s), automated incubation system(s), and high-resolution imaging of agar plates. More recently, artificial intelligence (AI) and interpretive algorithm software have been incorporated into these workflows, facilitating the automated reading of urine samples or the screening of multidrug-resistant bacteria. These systems can rapidly sort negative plates, perform automated colony counts, and analyze colony color on positive agar plates, enabling detection of polymicrobial growth. These innovations have facilitated 24/7 operations, replaced batch-based plate reading with more continuous readings, thus reducing turnaround times and providing technologist time savings that can be redirected to other diagnostic methods (2–4).

However, these technologies are not affordable for all laboratories, especially in low- and middle-income countries. Even in high-income countries, the colony transfer on MALDI-TOF target mainly remains a manual task, despite the emergence of automated deposition modules, such as Copan’s Colibri and BD Kiestra IdentifA, that allow to automate picking and deposition of bacterial colonies (2). Moreover, interpretation of digital plate images continues to require trained personnel, who may not be available around the clock. In addition, many public or private laboratories are experiencing a shortage of qualified technologists and face difficulties staffing evening and night shifts (1, 5). In this context, an affordable and fully automated method of identifying bacterial species directly on agar plates without manual intervention would provide substantial clinical and operational benefits.

Digital in-line holography (DIH) offers a promising approach to address this challenge. A high-resolution holographic image (hologram) encodes the three-dimensional object’s complex structure of an object within a 2D image (diffractogram). Using partially coherent illumination and a complementary metal-oxide-semiconductor (CMOS) sensor, holographic images of microbial colonies can be obtained directly through the agar plate. When a Petri dish containing colonies is positioned between the light source and the CMOS sensor, the recorded signal corresponds to a diffractogram or hologram of the colonies. Importantly, phase objects, such as bacterial colonies, can be imaged with good contrast.

This study aims at presenting a thorough proof of concept for a rapid, fully automated, non-destructive, and label-free method for bacterial identification at the species level, directly on agar plates. The approach combines DIH with convolutional neural networks (CNN) algorithms. Unlike standard 2D imaging, digital holography captures phase information related to optical path length, which depends on colony thickness, refractive index, and internal structure. This provides additional quantitative information beyond classical colony morphology, including features related to geometry, optical density, and potentially molecular composition. These additional dimensions may help distinguish bacterial species whose colonies appear similar in conventional images. We hypothesized that analysis of holograms of growing bacterial colonies by CNN inference could enable species-level bacterial identification. Building on previous works, we developed and evaluated the BAIO-DX prototype—an automated platform capable of processing standard 90 mm brain heart infusion (BHI) agar plates (6). The primary objective of this study was to construct an initial holographic image database using the BAIO-DX prototype, develop and train the AI algorithms, and evaluate the performance of this innovative identification method using clinical bacterial strains.

## MATERIALS AND METHODS

### Clinical samples, bacterial strains, and sample preparation

We used only clinical strains for this study. Monomicrobial cultures of clinical strains were collected or produced from two different sources. A total of 104 monomicrobial positive blood cultures (PBCs) were prospectively collected between 1 August 2022 and 31 January 2023. In addition, 469 clinical strains isolated from various clinical samples and representing 19 different species were obtained from the bacterial clinical strain collection of the Bacteriology Laboratory, Grenoble Alpes University Hospital. Written informed consent was not required in accordance with national regulations and institutional policies. As part of the standard of care (SoC) diagnosis procedure of bacteremia, an aliquot of each PBC bottle (BD BACTEC aerobic/F, Becton Dickinson, Pont de Claix, France) was transferred into a dry tube (BD Vacutainer) and diluted 1:50 in 5 mL sterile saline solution (BD). When SoC identification by MALDI-TOF mass spectrometry (performed on a Microflex LT instrument—Bruker Daltonics, Bremen, Germany—using MBT IVD software version 4.2.90 with database V10 DB-9468 MSP) confirmed a monomicrobial culture, 100 µL of the bacterial suspension was plated on brain heart infusion agar plates (BHI agar, Becton Dickinson) using the BD Kiestra Inoqula sample processor with the InocStreak S200 zigzag streaking pattern (7).

**Fig 1:**
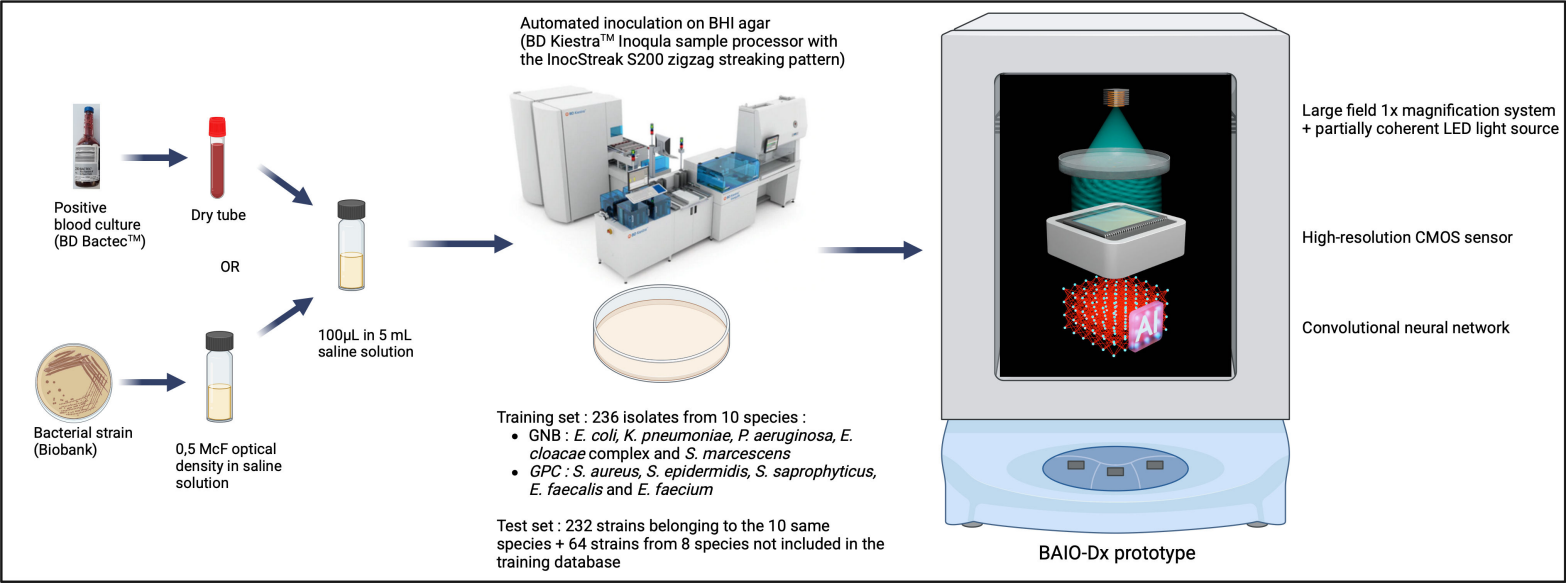
Schematic overview of the BAIO-DX digital inline holography system and study workflow. The diagram illustrates the experimental and analytical pipeline used for non-destructive, label-free bacterial identification directly on BHI agar plates. Clinical strains or monomicrobial positive blood cultures were plated and incubated within the BAIO-DX prototype, which integrates within an incubator a robotic arm and a digital inline holography imaging system. At 30-min intervals over an 18-h period, high-resolution holographic images of growing colonies were acquired using a large field 1× magnification setup with a partially coherent LED light source and a high-resolution CMOS sensor. Colonies were detected and segmented, and time-lapse image stacks were processed by convolutional neural network algorithms trained on a large database of annotated holograms to predict bacterial species.

Biobank strains were grown overnight on BHI agar plates at 35°C. Then, colonies were suspended in saline solution to a turbidity of 0.5 McFarland, diluted 1:50 in 5 mL saline solution, and plated using the same automated process. After inoculation, BHI plates were immediately placed in the BAIO-DX prototype for incubation (at 37°C, ambient atmosphere—no CO_2_ enrichment) and imaging (Fig. 1).

### Digital holographic imaging

DHI was performed using the BAIO-DX system (Fig. 1). High-resolution holograms (9,568 × 6,380 pixels) were acquired using a large-field 1× magnification optical system, a partially coherent LED light source (ILH-ON01-DEBL-SC211-WIR200, Intelligent LED Solutions), and a high-resolution monochromatic CMOS sensor (IMX 455 from Sony; 3.76 µm pixel pitch). Each 90 mm circular agar plate was scanned over the 24 × 36 mm sensor, generating 12 overlapping images per time point to cover the full surface, resulting in approximately 530 MPx per complete plate. Imaging was repeated every 30 min during an 18-h incubation. The system design was adapted from Wang et al., incorporating a larger CMOS sensor and an 18-plate storage capacity, allowing better automation for a higher throughput (6). Moreover, the BAIO-DX prototype has been designed to fit into a 115 L Binder incubator, providing enough space for the DHI system and the 18-shelf storage unit within the incubator. At each time point, a robotic arm retrieved plates from storage, removed their cover, and positioned them on the CMOS sensor for image acquisition, then returned them for continued incubation.

### Data processing and AI framework for bacteria identification

**Fig 2:**
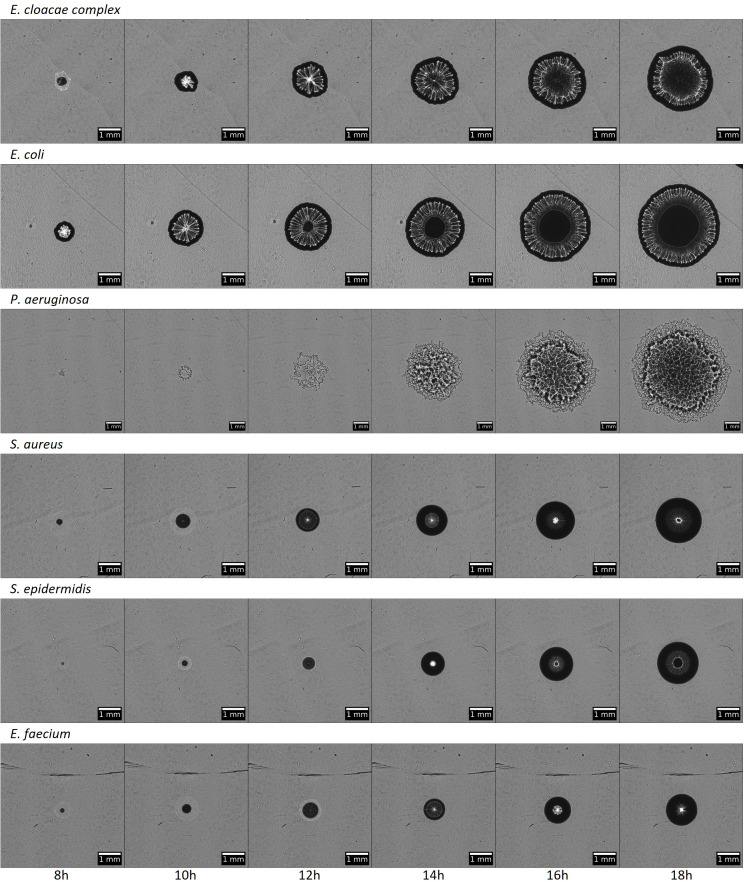
Time-lapse digital holographic images of a single bacterial colony every 2 h between 8 and 18 h for six different bacterial species. Representative digital holographic images of single colonies from six different bacterial species were acquired every 2 h from 8 to 18 h of incubation on BHI agar plates. These time-lapse image sequences served as the input data for a convolutional neural network-based species identification.

The 12 holograms obtained at each time point were stitched to form a single 530 MPx image of the full plate. Temporal alignment of images enabled colony tracking over time. Colonies were detected using classical segmentation algorithms, such as thresholding, region-growing, or edge detection, to separate individual colonies from the background and from adjacent colonies, and up to 200 isolated colonies per plate were selected for analysis. For each selected colony, a 36-frame stack (125 × 125 pixels) centered on the colonies was extracted (Fig. 2). These image stacks were used as input for a CNN architecture incorporating three-dimensional convolutional layers and spatially-adapted squeeze-excitation blocks (8–10). The training set consisted of 276 plates representing 10 bacterial species. Model training was performed 20 times using different random splits (70% training, 30% validation) to generate 20 independent models.

### Performance assessment of BAIO-DX system

Performance evaluation was carried out using an independent data set of 296 clinical strains and PBC samples. For each colony, inference was performed using all 20 CNN models, and the species with the highest consensus score was selected as the predicted identification. All 20 models are used for every prediction. The CNN prediction is done at the colony level. Briefly, for each colony, each model returns a SoftMax array consisting of a vector of 10 values. Each value is interpreted as the probability for the colony analyzed to belong to one of the ten species of the database. The output of the aggregated colony model is obtained by taking the average value of the outputs from the individual colony models. Then, the identification is performed at the dish level by averaging the outputs of the aggregated colony model for all colonies within a dish. Confidence scores ranged from 0 to 1; species-level identifications with scores <0.8 were rejected (arbitrary cut-off). Predictions were additionally classified according to a phylogenetic hierarchy. The CNN models generated confidence scores at multiple taxonomic levels (species, genus, family, order, class, phylum, or domain). The identification prediction was performed by browsing the tree from the root, following the nodes with the highest confidence until the score falls below the chosen threshold. The last node on the path with a score ≥threshold was reported as the final identification. Thresholds were optimized on the test data set to maintain ≥97% accuracy at each level. See supplemental material for additional details.

### Statistics

Identification results from the BAIO-DX system were compared to SoC identification results. We calculated positive percent agreement (PPA) of the innovative method as follows: PPA (%) = 100 × number of strains matching the identification obtained by SoC method/total number of strains tested. Performance of the method is also presented as a confusion matrix of the identification results.

## RESULTS

### Non-destructive label-free automated bacterial identification at the species level

The BAIO-DX system (Fig. 1) generated high-resolution digital holographic images that captured both the morphological and optical evolution of bacterial colonies during an 18-h incubation period, revealing species-specific growth dynamics and structural features (Fig. 2). On average, 179 ± 42 individual colonies were analyzed per BHI agar plate for the 276 monomicrobial samples representing 10 bacterial species used to train the model, yielding a total of 49,490 individual colonies (Table 1).

**TABLE 1:** Origin and distributions of the clinical strains and clinical samples in the train and test data sets

	Strains												Samples			
	UTI		PBC		RS		BJI		Biopsy		Other		PBC		Total	
Bacterial species	Train (*n* = 92)	Test (*n* = 15)	Train (*n* = 98)	Test (*n* = 137)	Train (*n* = 4)	Test (*n* = 1)	Train (*n* = 6)	Test (*n* = 104)	Train (*n* = 0)	Test (*n* = 3)	Train (*n* = 6)	Test (*n* = 2)	Train (*n* = 70)	Test (*n* = 34)	Train (*n* = 276)	Test (*n* = 296)
GNB																
*Escherichia coli*	0	0	22	20	0	0	0	2	0	0	0	1	13	5	35	28
*Klebsiella pneumoniae*	10	0	13	16	0	0	0	6	0	1	0	0	5	2	28	25
*Enterobacter cloacae* complex	9	2	9	9	0	0	0	14	0	0	0	0	6	1	24	26
*Serratia marcescens*	3	1	6	9	2	0	1	7	0	0	1	0	2	0	15	17
*Pseudomonas aeruginosa*	12	0	11	9	0	0	0	13	0	0	0	0	7	2	30	24
*Klebsiella aerogenes^a^*	0	0	0	5	0	1	0	2	0	0	0	0	0	0	0	8
*Klebsiella oxytoca^a^*	0	0	0	9	0	0	0	4	0	0	0	1	0	0	0	14
GPC																
*Staphylococcus aureus*	13	0	13	17	2	0	0	2	0	0	1	0	17	11	46	30
*Staphylococcus epidermidis*	3	0	12	17	0	0	4	6	0	0	1	0	13	6	33	29
*Staphylococcus saprophyticus*	12	12	0	0	0	0	0	0	0	0	0	0	0	0	12	12
*Staphylococcus lugdunensis^a^*	0	0	0	0	0	0	0	25	0	1	0	0	0	1	0	27
*Staphylococcus haemolyticus^a^*	0	0	0	2	0	0	0	5	0	0	0	0	0	0	0	7
*Staphylococcus hominis^a^*	0	0	0	0	0	0	0	0	0	0	0	0	0	3	0	3
*Staphylococcus capitis^a^*	0	0	0	0	0	0	0	2	0	0	0	0	0	0	0	2
*Staphylococcus caprae^a^*	0	0	0	0	0	0	0	1	0	0	0	0	0	0	0	1
*Staphylococcus cohnii^a^*	0	0	0	0	0	0	0	1	0	0	0	0	0	0	0	1
*Staphylococcus pettenkoferi^a^*	0	0	0	0	0	0	0	1	0	0	0	0	0	0	0	1
*Enterococcus faecalis*	16	0	8	16	0	0	0	8	0	1	1	0	6	0	31	25
*Enterococcus faecium*	14	0	4	8	0	0	1	5	0	0	2	0	1	3	22	16

^*a*^ Species not included in the training data set. GNB, Gram-negative bacteria; GPC, Gram-positive cocci; UTI, urinary tract infection; PBC, positive blood culture; BJI, bone and joint infection; RS, respiratory sample.

**Fig 3:**
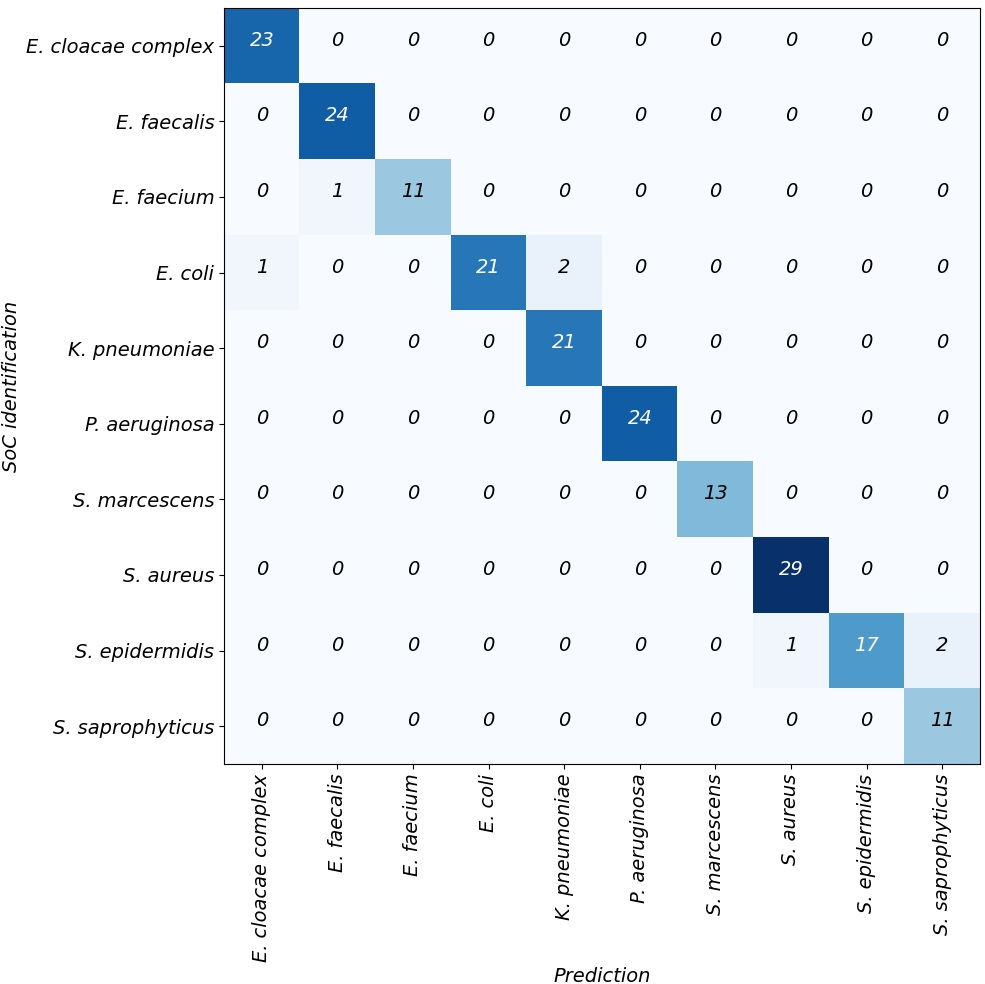
Confusion matrix of bacterial species identification using the BAIO-DX system and the phylogenetic thresholds. Confusion matrix comparing species-level predictions made by the BAIO-DX system to the reference identifications obtained by MALDI-TOF mass spectrometry for the 201 clinical isolates tested, belonging to the species included in the training data set and passing the species-level confidence threshold. Correct predictions appear along the diagonal, while off-diagonal values indicate misidentifications. The matrix illustrates the overall accuracy and the distribution of classification errors, demonstrating high concordance between the automated holography-based approach and SoC methods.

The performance of the BAIO-DX system was subsequently evaluated on 232 independent isolates belonging to the 10 same species (Table 1). The system identified 201/232 (86.6%) isolates at the species level, achieving a PPA of 96.5%. The remaining 31/232 (13.4%) isolates had confidence scores below the species-level threshold and were therefore not assigned a definitive identification. The confusion matrix of the bacterial identifications that passed the confidence threshold for species-level identification is presented in Fig. 3. It evaluates both the accuracy of the identification results and the confusion between the predicted and the true identification. Applying phylogenetically informed confidence thresholds at successive taxonomic levels (Fig. S1 to S3) substantially reduced misclassification errors—from 9.9% to 3.5%—while slightly decreasing the proportion of valid species-level identifications (Fig. S1).

### Identification at a higher taxonomic level is possible for strains below confidence threshold at the species level

Among the 31 isolates with confidence scores below the species-level threshold, the hierarchical phylogenetic approach enabled reliable identification at higher taxonomic ranks. Specifically, among the 31 strains not identified at the species level, 15/31 (48%) and 8/31 (26%) of the rejected samples at the species level were accurately identified at the genus or family level, respectively (Table 2), while 7/31 (23%) and 1/31 (3%) were only identified at the order or class level, all with 100% PPA. These results demonstrate that the model’s phylogenetically guided decision framework effectively mitigates erroneous classifications by promoting accurate identifications at a higher taxonomic level when species-level confidence is insufficient.

**TABLE 2:** Highest phylogenetic level of identification provided by BAIO-DX system for the 232 isolates tested (belonging to the 10 species of the training data set)

	Number of strains (%)				
	Species	Genus	Family	Order	Class
GNB					
*E. coli*	24 (86)	0	3 (11)	1 (3)	0
*K. pneumoniae*	21 (84)	1 (4)	3 (12)	0	0
*E. cloacae* complex	23 (88)	0	2 (8)	1 (4)	0
*S. marcescens*	13 (76)	1 (6)	0	3 (18)	0
*P. aeruginosa*	24 (100)	0	0	0	0
GPC					
*S. aureus*	29 (97)	1 (3)	0	0	0
*S. epidermidis*	20 (69)	7 (24)	0	1 (3.5)	1 (3.5)
*S. saprophyticus*	11 (92)	1 (8)	0	0	0
*E. faecalis*	24 (96)	0	0	1 (4)	0
*E. faecium*	12 (75)	4 (25)	0	0	0
Total (%)	201 (86.6)	15 (6.5)	8 (3.4)	7 (3.0)	1 (0.4)

### System performance on species absent from the training set

To assess the system’s generalizability, we also evaluated the accuracy of this first version of the BAIO-DX solution against an “unknown” subset of strains comprising 64 isolates from 9 species not included in the training set. These species belonged to two genera represented during training—42 coagulase-negative *Staphylococcus* and 22 *Klebsiella* isolates (Table 1; Tables S1 and S2). Although multiclass AI systems inherently attempt to assign each sample to the most probable known class, the application of phylogenetic confidence thresholds limited incorrect species-level assignments to 19/64 (29.7%) isolates. The identification of most of the strains was rejected at the species level but correctly identified at higher taxonomic levels. The system achieved excellent accuracy, correctly identifying 15/16 (94%), 7/7, and 15/15 strains at the genus, family, or order levels, respectively.

## DISCUSSION

In this study, we developed and evaluated a novel automated, non-destructive, and label-free system for bacterial colony identification directly on agar plates and without any human intervention. The BAIO-DX system combines digital in-line holography with convolutional neural network algorithms to achieve species-level identification. Using a large training data set comprising strains from 10 of the most frequently isolated bacterial species in clinical microbiology (accounting for ≥65% of positive blood cultures and ≥90% of urine samples [11]), the first version of BAIO-DX correctly identified 82% of monomicrobial samples containing one of these species. For 13.4% of isolates, species-level identification did not reach the predefined confidence threshold. Importantly, among these isolates, 48%, 26%, and 23% were correctly assigned to the genus, family, or order level, respectively, without any misidentification. All *Staphylococcus aureus*, *Klebsiella pneumoniae*, *Enterococcus faecalis*, *Enterobacter cloacae* complex, *Serratia marcescens*, and *Staphylococcus saprophyticus* isolates that reached the confidence threshold were correctly identified at the species level. Misidentifications were rare (3%) and involved uncommon isolates of *Escherichia coli*, *Staphylococcus epidermidis*, or *Enterococcus faecium*. Collectively, these results provide a robust proof of concept that high-resolution holographic signatures—reflecting colony morphology and physicochemical properties, such as refractive index, geometry, and molecular composition—can support accurate bacterial species identification. To our knowledge, this level of performance has not previously been achieved for the simultaneous analysis of 10 bacterial species using holographic imaging-based approaches. Earlier studies relied on dedicated agar pads that differ substantially from routine clinical agar plates or on chromogenic media that are either species-restricted or associated with increased costs (12, 13). In contrast, we selected a translucent, nutrient-rich BHI agar that supports the growth of most clinically relevant bacterial pathogens. It should be noted that as experiments were conducted over an extended period and therefore involved several media lots. Thus, potential lot-to-lot variability was inherently taken into account during the training process and reflected in the identification results. Although the use of BHI agar is not very frequent in routine clinical microbiology currently, because pathogenic bacteria detection relies on phenotypic characteristics (e.g., hemolysis) requiring other media, the use of BHI broth is common. New agar media have emerged over time to improve clinical microbiology diagnosis. Furthermore, the fully automated data acquisition implemented in BAIO-DX enabled the analysis of a substantially larger number of colonies than in previous reports, facilitating the generation of a large, diverse training data set that is essential for robust CNN performance (6, 12, 14–22). An incubation time of 18 h was selected as a compromise between sufficient colony size for robust digital holographic analysis and an incubation time resembling current clinical laboratory practices (most agar plates are visualized or pictured once a day) but that may reduce time-to-result thanks to our fully automated process with no additional intervention required. Future work will investigate whether similar performances can be achieved with shorter incubation times. Unlike standard multiclass artificial intelligence approaches, which systematically assign an isolate to a species regardless of uncertainty, our system integrates prediction scores and confidence thresholds across multiple phylogenetic levels. This strategy improved overall accuracy while allowing reporting at higher taxonomic ranks when species-level confidence was insufficient. Such partial identifications may still be clinically valuable, for example, to guide early antimicrobial therapy or to select appropriate antibiotic susceptibility testing (AST) panels. However, although the confidence threshold provided a useful reliability filter for the trained species, it was not specifically optimized for out-of-distribution detection. Future work will evaluate more conservative thresholds, including an “unknown” class, to improve the rejection of organisms outside the training universe.

The accuracy of this first version of the BAIO-DX solution was also evaluated for 64 isolates from 9 species not present in the training set but belonging to two bacterial genus included in the training set (42 coagulase-negative *Staphylococcus* and 22 *Klebsiella* strains). Overall, 17/64 (27%) of the strains were incorrectly identified at the species level. Nine *Staphylococcus* isolates belonging to species not present in the training set were predicted as *S. epidermidis,* and two *S*. *hominis* isolates as *S. aureus* (Table S1). Moreover, one *S*. *lugdunensis* isolate was identified as *E. faecium*. Among the *Klebsiella* genus group, four isolates belonging to species not present in the training set were identified as *K. pneumoniae,* and three as *E. coli*. Moreover, 16/64 (25%) strains were identified only at the genus level by the system, with one misidentification. One *K. aerogenes* isolate was identified as belonging to the *Escherichia* genus. All other strains were correctly predicted but at higher taxonomic levels: 7 *Klebsiella* isolates were predicted as belonging to the *Enterobacteriaceae* family, respectively, 9 *Staphylococcus* and 6 *Klebsiella* isolates were predicted as belonging to *Bacillales* and *Enterobacterales* order; and 7 *Staphylococcus* were predicted in the *Bacilli* class.

The main limitation of this study is the restricted number of species included in the training data set, although this number exceeds that of most previously published studies. Moreover, we only tested a few additional species not present in the training data set to see the behavior of the system, but a more thorough specificity study will be required in the next steps of development of this approach. In addition, the technology requires translucent culture media; while BHI agar supports the growth of most pathogens, it does not encompass all clinically relevant species. Only aerobic growth conditions were evaluated, and results were interpreted at the agar plate level by aggregating all isolated colonies from monomicrobial samples. We did not test mixed samples at this step of development. We will have to challenge this technology in the future with high-volume specimens like urine, wounds, or respiratory samples. Moreover, this was a single-center study using a single prototype, and larger multicenter evaluations with multiple instruments will be required to assess robustness and generalizability. Our study was, at this step, only designed to assess a thorough proof of concept, and much work will have to be done before having an automaton approved for clinical microbiology use. Several methodological strengths mitigate these limitations. To minimize plate-related bias, data splitting was performed at the plate level: all colonies from a given plate were assigned exclusively to either the training or the test set. This approach preserved data independence and reduced the influence of dish-specific factors and strain-related correlations, which are often overlooked in similar studies (6, 12, 13). While system accuracy could be increased (up to approximately 99%) by raising confidence thresholds, this would be at the expense of a higher rejection rate. Conversely, colony-level identification—although less accurate—may ultimately allow analysis of polymicrobial samples. In the prototype used in this study, plates remained inside the incubator during image acquisition, avoiding repeated manual interruption of incubation. Images were acquired every 30 min to capture growth dynamics with sufficient temporal resolution during this proof-of-concept phase. The system currently requires multiple acquisition time points to achieve optimal performance, generating large volumes of data, as previously reported (12, 15); however, reducing acquisition frequency to every 2–3 h appears feasible with minimal impact on accuracy. Future work will evaluate whether less frequent imaging can preserve identification performance while reducing data volume and acquisition complexity. Finally, by challenging the system with isolates not included in the training database, we demonstrated that the phylogenetic prediction strategy markedly reduces misidentification compared with conventional multiclass classifiers.

Compared with current bacterial identification workflows based on MALDI-TOF mass spectrometry, which depend on both bacterial growth and the availability of trained personnel, BAIO-DX can provide real-time identification after 18 h of incubation without manual intervention. The non-destructive, reagent-free nature of this technology, combined with relatively low automation costs, may facilitate implementation in laboratories with limited resources, including those in low- and middle-income countries. The system appears well suited for integration into existing total laboratory automation (TLA) platforms and may help reduce the number of culture media required for certain specimens or assist in the automated selection of AST panels.

In conclusion, this study introduces a promising, fully automated, non-destructive approach to bacterial identification directly from agar plates. Although substantial development is still required—particularly to expand the reference database and species diversity—these results support the feasibility of clinical implementation. Future work should focus on targeted expansion of species relevant to specific specimen types (e.g., urine), shortening the time to identification (e.g., 8 h), and improving performance through multimodal approaches that integrate Gram stain data, conventional plate imaging from TLA systems, or modified culture media incorporating selective or chromogenic additives. Beyond clinical microbiology, this technology may also find applications in environmental monitoring, food and water safety, and pharmaceutical sterility testing.

## Data Availability

The data sets generated and analyzed during the current study consist of high-volume imaging data and associated metadata. Due to technical storage limitations and third-party ownership restrictions, the full data sets cannot be made publicly available through an open repository. The data are subject to institutional and industrial confidentiality agreements, and the authors are therefore not authorized to provide unrestricted public access to the complete raw image database. However, representative data supporting the findings of this study, including selected image data sets and associated metadata, may be made available from the corresponding author upon reasonable request and subject to authorization from the data owner.

## SUPPLEMENTAL MATERIAL

Supplemental material spectrum.00080-26-s0001.docx Fig. S1 to S3; Tables S1 and S2.

ASM does not own the copyrights to Supplemental Material that may be linked to, or accessed through, an article. The authors have granted ASM a non-exclusive, world-wide license to publish the Supplemental Material files. Please contact the corresponding author directly for reuse.
