# Supplementary material for: Non-destructive label-free automated identification of bacterial colonies at the species level directly on agar media using digital holography and convolutional neural network algorithms
Source: Microbiol Spectr. 2026 Jul 13;14(8):e00080-26. doi: 10.1128/spectrum.00080-26 (PMC13436200; doi:10.1128/spectrum.00080-26)
Supplement: Supplementary file 1 [file spectrum.00080-26-s0001.docx]

**Online Supplement to:**

**Non-destructive label-free automated identification of bacterial colonies at the species level directly on agar media using digital holography and convolutional neural network algorithms**

**Prisca Perlemoine, Jordan Belissard, Bruno Burtschell, Nassim Halli, Luc Martin, Camille Brunet, Maxime Gougis, Patrick Schiavone, Yvan Caspar**

[Supplementary methods	2](#_Toc202819406)

[Identification using phylogenetic information	2](#_Toc202819407)

[Results	3](#_Toc202819408)

[Identification performances without the phylogenetic approach and the confidence thresholds at each taxonomic level	3](#_Toc202819409)

[Figure S1: Confusion matrix of bacterial species identification using the BAIO-DX system but not the phylogenetic thresholds	3](#_Toc202819410)

[Table S1: Detailed predictions for the 42 tested strains belonging to *Staphylococcus* genus but to species not present the training set	4](#_Toc202819411)

[Table S2: Detailed predictions for the 22 tested strains belonging to *Klebsiella* genus but to species not present the training set	4](#_Toc202819412)

[Identification using sample rejection	5](#_Toc202819413)

[Figure S2: Identification of a strain of *S. epidermidis* identified as *Staphylococcus* sp using the BAIO-DX system and the phylogenetic prediction thresholds	6](#_Toc202819414)

[Figure S3: Identification of a strain of *S. capitis* using the BAIO-DX system and the phylogenetic prediction thresholds	7](#_Toc202819415)

[References	7](#_Toc202819416)

# Supplementary methods

## Identification using phylogenetic information

Each bacterial strain of the test database was placed in a subset of the phylogenetic tree in which each level of depth represents a different taxon (respectively species, genus, family, order, class, phylum, and domain) according to the taxonomic information of the corresponding species retrieved from NCBI database [1].

The neural network models output a list of SoftMax values for each sample. Each element of the list corresponds to a different class in the classifier. While in a standard approach we only used the maximum value of the SoftMax array as the prediction score, in the phylogenetic approach, the SoftMax values are used as the probability of the sample to belong to the corresponding species and are assigned to the leaf nodes of the tree. For each node at a higher level of the phylogenetic tree which represents an upper-level taxon, we calculated a score based on the sum of the scores of its children. By construction, as the sum of the SoftMax values is 1, the root node of the tree has a score equal to 1.

The identification prediction is performed by browsing the tree from the root, following nodes until the score falls below a chosen threshold. The last node on the path with a score above the chosen threshold is considered the prediction. To ensure satisfactory accuracy at each taxonomic level, level-specific thresholds were fitted to achieve the targeted accuracy.

The final phylogenetic prediction may then be obtained not at the species level but at a higher taxonomic level for each strain tested based on these thresholds.

# Results

## Identification performances without the phylogenetic approach and the confidence thresholds at each taxonomic level

Figure S1: Confusion matrix of bacterial species identification using the BAIO-DX system but not the phylogenetic thresholds**.** Confusion matrix comparing species-level predictions made by the BAIO-DX system to the reference identifications obtained by MALDI-TOF mass spectrometry for the 232 clinical isolates tested belonging to the species included in the training dataset. Correct predictions appear along the diagonal, while off-diagonal values indicate misidentifications. The matrix illustrates the overall accuracy and the distribution of classification errors, demonstrating high concordance between the automated holography-based approach and standard-of-care (SoC) methods. Without the phylogenetic thresholds, the systems provides an identification at the species level for all strains tested but with an overall accuracy of only 90.1%.

## Table S1: Detailed predictions for the 42 tested strains belonging to *Staphylococcus* genus but to species not present the training set

|  | | **Prediction by taxonomic levels (nb of strains)** | | | | | | | |
| --- | --- | --- | --- | --- | --- | --- | --- | --- | --- |
|  |  | **Class** | **Order** | **Genus** | **Species** | | | | |
|  |  | *Bacilli* | *Bacillales* | *Staphylococcus* | *S.aureus* | *S. epidermidis* | | *E. faecium* |  |
| **SoC**  **identification** | *S. lugdunensis* | 6 | 9 | 8 |  | 3 | 1 | | |
|  | *S. hominis* | 1 |  |  | 2 |  |  | | |
|  | *S. haemolyticus* |  |  | 4 |  | 3 |  | | |
|  | *S. caprae* |  |  |  |  | 1 |  | | |
|  | *S. pettenkoferi* |  |  |  |  | 1 |  | | |
|  | *S. cohnii* |  |  |  |  | 1 |  | | |
|  | *S. capitis* |  |  | 2 |  |  |  | | |

## Table S2: Detailed predictions for the 22 tested strains belonging to *Klebsiella* genus but to species not present the training set

|  | | **Prediction by taxonomic levels (nb of strains)** | | | | | |
| --- | --- | --- | --- | --- | --- | --- | --- |
|  |  | **Order** | **Family** | **Genus** | | **Species** | |
|  |  | *Enterobacterales* | *Enterobacteriaceae* | *Escherichia* | *Klebsiella* | *E. coli* | *K. pneumoniae* |
| **SoC**  **identification** | *K. aerogenes* | 1 | 4 | 1 |  | 1 | 1 |
|  | *K. oxytoca* | 5 | 3 |  | 1 | 2 | 3 |

## Identification using sample rejection

For this study the results are presented with a 13.4% rejection ratio to achieve an accuracy of 96.8%. This corresponds to a 0.82 rejection score at species level.

Figure S2: Identification of a strain of *S. epidermidis* identified as *Staphylococcus* sp using the BAIO-DX system and the phylogenetic prediction thresholds**.** Figure S2 illustrates an example of an identification using the BAIO-DX system and the phylogenetic prediction approach, with species level threshold set to 0.82. This example corresponds to a strain of *S. epidermidis* misidentified as *S. saprophyticus* at species level but with a prediction score of only 0.62 leading to rejection of the identification at the species level. It is however identified as *Staphylococcus* sp at the genus level with a confidence score of 0.98 superior to the genus threshold*.* If we were to use the classical prediction approach with threshold only at the species level, this sample would have been rejected as the score of the higher ranked species is lower than the 0.82 threshold, and no information would have been provided for this strain. This demonstrates the usefulness of the phylogenetic prediction approach in cases where species-level identification might be challenging.

Figure S3: Identification of a strain of *S. capitis* using the BAIO-DX system and the phylogenetic prediction thresholds**.** Figure S3 illustrates an example of an identification using the BAIO-DX system and the phylogenetic prediction approach, with each taxonomic-level threshold set to 0.8. This example corresponds to a strain of *Staphylococcus capitis*, a species that is not present in the training dataset. Although the model is not trained on the tested species, the system is able to reject the identification at the species level (highest confidence score = 0.61) but to classify the strain as belonging to the *Staphylococcus* genus (confidence score = 1).

# References

[1]	National Library of Medicine : Taxonomy Database
